# Association of physical exercise and calcium intake with bone mass measured by quantitative ultrasound

**DOI:** 10.1186/1472-6874-10-12

**Published:** 2010-04-07

**Authors:** Yannis Dionyssiotis, Ioanna Paspati, Georgios Trovas, Antonios Galanos, Georgios P Lyritis

**Affiliations:** 1 Laboratory for Research of the Musculoskeletal System, University of Athens, Kifissia, Greece; 2 Rehabilitation Department, Rhodes General Hospital, Rhodes, Greece

## Abstract

**Background:**

Interventions other than medications in the management of osteoporosis are often overlooked. The purpose of this study was to investigate the association of physical activity and calcium intake with bone parameters.

**Methods:**

We measured the heel T-score and stiffness index (SI) in 1890 pre- and postmenopausal women by quantitative ultrasound (QUS) and assessed physical activity and dietary calcium intake by questionnaire. Participants were divided according to their weekly physical activity (sedentary, moderately active, systematically active) and daily calcium consumption (greater than or less than 800 mg/day).

**Results:**

SI values were significantly different among premenopausal groups (p = 0.016) and between sedentary and systematically active postmenopausal women (p = 0.039). QUS T-scores in systematically active premenopausal women with daily calcium intake > 800 mg/day were significantly higher than those in all other activity groups (p < 0.05) independent of calcium consumption.

**Conclusions:**

Systematic physical activity and adequate dietary calcium intake are indicated for women as a means to maximize bone status benefits.

## Background

Osteoporosis, a systemic skeletal disease characterized by low bone mass and micro architectural deterioration of bone tissue leading to increased fragility [1], is a worldwide public health problem. Approximately 40-50% of Caucasian women will suffer an osteoporosis-related fracture in their lifetime [2]. There has been significant progress in the pharmacological treatment of osteoporosis, and although extensive research in this area continues, there is also a concerted effort to develop preventive strategies and identify populations with low bone mass and more than one risk factor for an osteoporotic fracture [3,4]. In addition to medications and other interventions, physical activity is an important factor for bone health that is often overlooked. Physical activity increases bone mass during childhood and adolescence, and is necessary to achieve the highest peak bone mass [5-7]. Especially in postmenopausal individuals, the results of studies on the positive association of physical activity with bone status are conflicting [8,9]. However, it is clear that physical activity is vital in adults [10,11] because it reduces the rate of bone loss during the peri-menopausal period, and decelerates bone loss associated with aging [8-12]. Studies on the association of exercise plus calcium intake and bone mass have also yielded contradictory results, due in part to differences in methodology and/or study design [13,14]. Neither exercise nor calcium supplementation alone have proven effective in compensating for estrogen-deficient bone loss. Nonetheless, there have been studies that provide support for the idea that physical activity and dietary calcium intake interact to produce a positive effect on bone mineral density (BMD) [15-18].

In a prospective study, Prince et al. showed that the addition of an exercise regimen incorporating weight-bearing exercises was necessary to achieve beneficial effects of calcium supplementation on femoral neck BMD in young postmenopausal women [13]. In a meta-analysis of 17 exercise trials, Specker concluded that the beneficial effects of exercise on bone mass were only realized if calcium consumption was high, suggesting a synergistic action of calcium and exercise [19].

As a means to evaluate bone status, quantitative ultrasound (QUS) devices have both advantages and limitations. Frost et al. concluded that the current World Health Organization criteria for the diagnosis of osteoporosis in postmenopausal women cannot be applied to calcaneal QUS measurements [20]. Given the low cost and portability of QUS equipment, the International Quantitative Ultrasound Consensus Group has recommended QUS for bone mass screening [21]. Most population studies that have investigated the effect of physical activity on BMD have used the dual energy X-ray absorptiometry (DXA) method, whereas studies based on QUS measurements are less common. Although there are limited data regarding the effects of exercise on calcaneus ultrasound parameters, the results that do exist indicate that QUS parameters measured at the heel may respond to physical activity [22-27]. The purpose of this cross-sectional study was to evaluate the association of physical activity and calcium intake, separately and together, on the calcaneus measured by QUS in the Greek female population.

## Methods

### Recruitment

During 2004 and 2005, 1890 women from 14 different municipalities of Greece ranging from the south (Attica county) to the north (Imathia county), including mainland Greece (Fthiotida and Eurytania counties), participated in a screening program for osteoporosis. Participants were stratified according to socioeconomic distribution and geographic locales, including urban and rural centers, countryside, mountainous and low-lying regions of the four counties. Subjects were recruited through the media and local women's societies. Because of the lack of BMD devices in many areas of Greece, the announcement by the media of a screening program for osteoporosis was a social event. Not only did women suffering from osteoporosis respond, but healthy women, young premenopausal women, and others who were curious about the disease and wished to learn more about it (including men) also participated freely in these events. Hence, we believe that our study population represents a randomly selected sample of Greek women. Examinations were performed free of charge by trained personnel and were supervised by medical doctors trained in the use of QUS devices. Based on responses to questionnaires, women suffering from any health condition known to affect bone metabolism (e.g., endocrine, renal, or bone diseases) or using any kind of bone-acting medication or calcium supplementation within 6 months were excluded. Women were weighed on a scale (SECA 760, Vogel & Halke GmbH & Co., Hamburg, Germany) and their height was measured with a portable wall-mounted ruler in the upright position (accuracy 0.1 cm). Body Mass Index (BMI) was calculated as weight in kilograms divided by height squared (m^2^).

### Protocol and Interview questions

This study was organized by the Hellenic Society of Osteoporosis Patients Support, and approved by the Ethics Committee of the Laboratory for Research of the Musculoskeletal System, University of Athens, where the data were analyzed. The protocol was designed according to the Declaration of Helsinki, and all subjects were informed about the study and gave written informed consent. We certify that all applicable institutional and governmental regulations concerning the ethical use of human volunteers were followed during the course of this research. The Hellenic Society of Osteoporosis Patients Support is a member of the International Osteoporosis Foundation and organizes programs to educate the population about osteoporosis and performs screening tests.

All women were interviewed in person using a structured survey questionnaire [Additional file 1] that provides socio-demographic data, and information about medical history, risk factors, nutritional habits, and physical activities. This questionnaire is a modification of the MEDOS study questionnaire, which has been translated into the Greek language and validated. It is adapted to the Greek population, taking into consideration the characteristics and life style habits of Greek people; it includes specific questions that assess the type and duration of physical activity (in hours) on a weekly basis and daily dietary calcium intake [28]. The question relating to physical activity was "How often do you participate in one or more physical activities of 20 to 30 minutes duration per session during the week?" The given answers were: not at all or less than once (1), once (2), two to three times per week (3), and more than three times per week (4). For the purposes of the analysis, women were classified into three groups according to their physical activity level: sedentary (one or no sessions per week [answer 1 or 2]), moderately active (two to three sessions per week [answer 3]) and systematically active (more than three sessions per week [answer 4]). Activities considered to satisfy the criteria of bone - promoting physical activities included gym exercises (e.g., walking on a treadmill, muscle strengthening, impact-aerobic exercises) as well as dancing and gardening. The common characteristics of all the above-mentioned activities are that they include loading, weight-bearing elements and muscular strengthening - factors considered to be the most important aspects of physical activity in the context of osteoporosis. We did not include housework as a qualifying activity because it does not provide benefits to bones. Dietary calcium intake in milligrams per day, measured at one time point, was calculated from the subjects' answers on the questionnaire using tables that define the calcium content of local foods. The section of the questionnaire related to calcium intake is a semi-quantitative food-frequency questionnaire. The questionnaire assesses the average frequency of calcium intake over the previous year. For each woman, we calculated calcium intake by multiplying the reported frequency of consuming each food item by the calcium content for the specified portion size. To determine calcium intake, we summed the calcium intake from the following items on the food frequency questionnaire: whole milk, skim or low-fat milk, yogurt, ice cream, feta cheese and other types of cheese, cruciferous vegetables, and nuts. Subjects were asked how often in the last year they usually ate each of these foods. Food models were used to assist subjects in rating their usual portion size for each food as small, medium, or large. Inclusion of foods in the questionnaire was based on their contribution to total calcium intake in European Union adults. The food-composition database used to calculate calcium values is based primarily on US Department of Agriculture publications supplemented with other Greek data in the literature and manufacturer's data [29]. Consumption of alcohol (frequency and amount) of all types during a week (portions/week), and cigarette-smoking habits (number of cigarettes/day) were also determined from the questionnaire.

### QUS measurements

QUS measurements of the calcaneus were performed on the non-dominant heel (one-leg study) of all study participants, with the subject in the sitting position, using a water-based Achilles Express ultrasonometer (GE Lunar Corp., Madison, WI, USA). The heel was placed between two ultrasonic transducers in a water bath at a temperature of 37°C. The ultrasound uses high-frequency sound waves to measure heel bone, measuring the velocity of the ultrasound signal (speed of sound [SOS]) and frequency attenuation (broadband ultrasound attenuation [BUA]). The Achilles Express ultrasonometer measures ultrasound variables of the os calcis to provide a clinical measure called the stiffness index (SI), which is calculated automatically from the combined SOS and BUA. This index, expressed as a percentage of the results from young adults (peak bone mass), was established by Lunar. BUA and SOS are given as absolute values, but the system provides no normative data for either parameter. Therefore, T-scores for BUA and SOS cannot be calculated [30,31]. The sonography-determined stiffness is automatically determined using the scanner software [32]. Stiffness is the default parameter used by the manufacturer for demographic comparison of patient data. All measurements were calculated using three identical Achilles Express devices by the same operators using the same ultrasonometers. The in vitro precision of the Achilles, estimated by measuring a phantom daily for 45 days, was 0.84% for BUA and 0.12% for SOS. The quality control procedure was performed each day before the in vivo measurements using the standard phantom. We assessed the in vivo short-term precision in 20 healthy volunteers, measured three times each. The coefficients of variation were 0.93% (± 0.21) for BUA and 0.15% (± 0.03) for SOS.

### Analysis

Because data about physical activity, calcium intake, and anthropometric and QUS parameters in the Greek population was lacking, the Greek Ministry of Health and regional counties authorized osteoporosis events as a way to obtain information about these variables in different areas of Greece. For this reason, we initially performed a simple analysis of all these variables according to the age decade of participants (see Results and Discussion). The main analysis of our study was based on menopausal status. Participants (mean age 57 ± 12 yrs) were divided into premenopausal (n = 446) and postmenopausal (n = 1444) women; the mean age of menopause was 48 ± 5 years. Of the study participants, 25% had a previous fragility fracture history, most of whom were postmenopausal women (p < 0.001); the age decade and associated fracture frequencies were < 50: 17%; 50-59: 21%; 60-69: 36%; 70-79: 48%; 80-89: 51%. Subjects were also divided in three groups according to weekly physical activity frequency as follows: sedentary group (n = 1710), moderately active group (n = 121), and systematically active group (n = 59). The anthropometric data for the total population (age decades, distribution of menopause status, and activity groups) are presented in Tables 1, 2, 3, 4. Premenopausal women with amenorrhea (> 6 months) or undergoing early menopause (< 40 years old) were excluded after the first analysis in order to further analyze an apparently healthy group. Participants were divided according to daily dietary calcium intake into a < 800-mg/day group (n = 1310, 69.3%) and a > 800-mg/day group (n = 567, 30.7%).

**Table 1:** Mean values ± sd and p-values of anthropometric measurements and QUS variables (T-score, stiffness index) in the different age-decades.

Women Variables	< 40 years	40-49 yrs	50-59 yrs	60-69 yrs	70-79 yrs	80+ yrs	p-value ANOVA
		
	n = 125	n = 321	n = 387	n = 493	n = 450	n = 114	
**Weight (Kg)**	68.4 ± 14.1	69.39 ± 14.3	72.45 ± 12.2	73.62 ± 13	70.83 ± 11.8	65.1 ± 10.3	< 0.001

**Height (m)**	1.65 ± 0.06	1.62 ± 0.07	1.59 ± 0.06	1.58 ± 0.06	1.55 ± 0.08	1.53 ± 0.08	< 0.001

**BMI (kg/m^2^)**	25.23 ± 5.14	26.6 ± 5.16	28.56 ± 4.83	29.71 ± 5	29.4 ± 5	28.7 ± 5	< 0.001

**T-score**	-0.1 ± 1.2	-0.3 ± 1.3	-0.9 ± 1.2	-1.5 ± 1.4	-2.1 ± 1.3	-2.5 ± 1.2	0.01

**Stiffness index**	97.62 ± 19	95.9 ± 16	88.64 ± 16	81.54 ± 16.7	75.34 ± 16.8	71.77 ± 18.7	< 0.001

Age decades 20-29 and 30-39 were grouped in one (< 40 yrs) because of the small numbers of participants compared to the other groups.

**Table 2:** This table presents the numbers of participants in each activity group and percent according to menopausal status and age category.

Total population	Premenopausal		Postmenopausal				
**Physical activity**	**20-39 yrs** **n = 77**	**40-49 yrs** **n = 120**	**40-49 yrs** **n = 221**	**50-59 yrs** **n = 383**	**60-69 yrs** **n = 493**	**70-79 yrs** **n = 450**	**80+** **yrs** **n = 114**

***sedentary***	55 (70%)	102 (85%)	188 (85%)	340 (89%)	453 (92%)	432 (96%)	112 (98.5%)

***moderately active***	15 (20%)	12 (10%)	22 (10%)	23 (6%)	31 (6.4%)	15 (3.4%)	2 (1.5%)
***active***	7 (10%)	6 (5%)	11 (5%)	20 (5.3%)	9 (1.7%)	3 (0.7%)	0%

Age decades 20-29 and 30-39 were grouped in one (< 40 yrs) because of the small numbers of participants compared to the other groups.

**Table 3:** Mean values ± sd and p-values of QUS T-score calculated according to daily calcium intake in premenopausal activity groups using 800 mg daily as cut off point

Parameters	Daily calcium intake	Physical Activity	QUS T-score	p-value
				
Participants				ANOVA
		sedentary	-0.4 ± 1.3	
			
premenopausal (n = 268)	*below* *800 mg*	moderate active	-0.2 ± 1.1	0.181
			
		vigorous active	0.3 ± 1.2	

		sedentary	-0.1 ± 1.3	
			
premenopausal (n = 152)	*above* *800 mg*	moderate active	0.1 ± 1.1	0.032
			
		vigorous active	0.9 ± 1.2	

Covariates appearing in the model are evaluated at the following values: BMI = 26.3, age = 42.5, (vigorous active vs. sedentary, p = 0.028)

**Table 4:** Mean values ± sd and p-values of QUS T-score calculated according to daily calcium intake in postmenopausal activity groups using 800 mg daily as cut off point

Parameters	Daily calcium intake	Physical Activity	QUS T-score	p-value
				
Participants				ANOVA
		sedentary	-1.6 ± 1.4	
			
postmenopausal (n = 887)	*below* *800 mg*	moderate active	-1.5 ± 1.6	0.696
			
		vigorous active	-1.4 ± 0.9	

	*above*	sedentary	-1.6 ± 1.4	
			
postmenopausal (n = 371)	*800 mg*	moderate active	-1.5 ± 1.6	0.229
			
		vigorous active	-0.9 ± 1.4	

Covariates appearing in the model are evaluated at the following values: BMI = 29.22, age = 64.8

All quantitative data are represented by the number of patients (n) and expressed as mean value (mean) and standard deviation (SD); qualitative data are expressed as the number (n) and percentage (%) of patients. Quantitative variables were analyzed using one-way and two-way analysis of variance (ANOVA) models. To compare QUS parameters across each of these 6 × 2 cross-classifications (age group × second factor), we performed a two-way ANOVA with interaction, including age group and the second factor, as main effects. Differences among the six groups were examined by a one-way ANOVA. When significant interactions did emerge, within group comparisons were also made using one-way ANOVAs and Bonferroni tests for pair-wise comparisons. Subjects were also classified into surrogate categories based on several conventionally accepted factors that might influence bone mass (e.g., normal or abnormal BMI, above or below a specific cutoff point for physical activity or calcium intake). Qualitative variables were analyzed using Chi-square (χ^2^) and Fisher's exact tests. Simple correlations were calculated using Pearson's method. A stepwise, forward, multiple-regression analysis was performed to determine the best predictors of SI parameters in all age groups; separate analyses were performed for women less than 50 years old and those more than 50 years of age. All tests were two-sided, and a p-value < 0.05 was considered statistically significant. Statistical analyses were performed using the statistical package SPSS (Statistical Package for the Social Sciences; SPSS Inc, Chicago, IL, USA) version 12.00 for Windows.

## Results

### General characteristics of the study population

**Figure 1:**
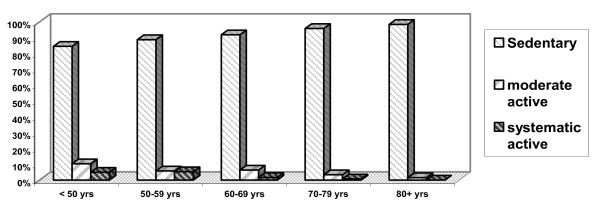
**Weekly physical activity according to age decade**. This figure depicts a categorization of weekly physical activity frequency according to age-decade *only*. The cut-off decade for reduction of physical activity in women was found 60-69 decade.

**Figure 2:**
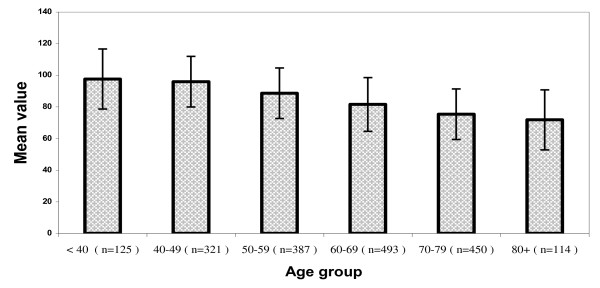
**Stiffness Index (SI) values in study's population**. This figure depicts a categorization of stiffness index values and number of participants in our study population according to age-decade *only*. No significant decline in stiffness index was seen in women (premenopausal and postmenopausal) below 50 years. This parameter exhibits a fall pattern especially after the 6^th ^decade as seen in the figure (p = 0.001).

**Figure 3:**
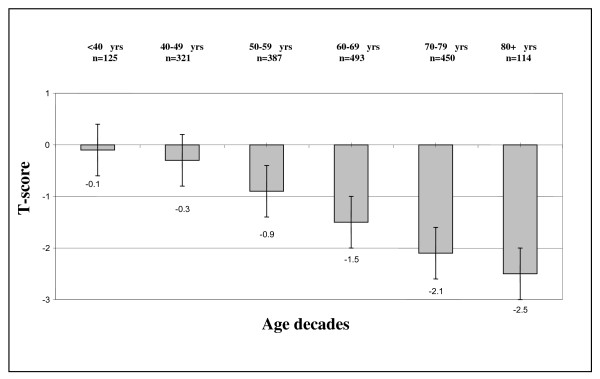
**Mean QUS T-score in various age decades**. This figure depicts a categorization of QUS T-score values and number of participants in our study population according to age-decade *only*. No significant decline in T-score was seen in women (premenopausal and postmenopausal) below 50 years. This parameter exhibits a fall pattern especially after the 5^th ^decade as seen in the figure (p = 0.01).

**Figure 4:**
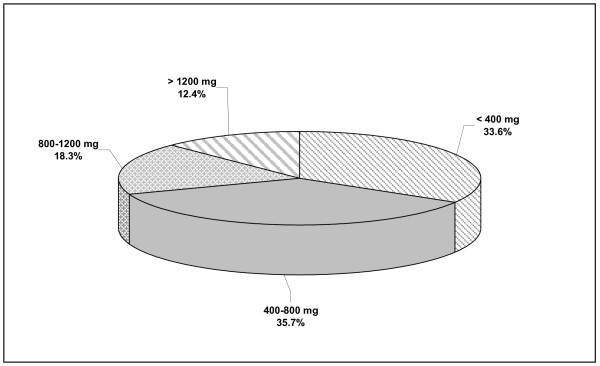
**Daily amount of dietary calcium intake in total population**. This figure depicts the daily amounts of dietary calcium intake (and percent) in total population.

Anthropometric measurements, T-scores and SI values for the study population of Greek women by age-decade groups (mean values ± SDs) are presented in Table 1 and Figures 1, 2, and 3. An analysis of the anthropometric data showed a linear decline in height during aging (p < 0.001). Body weight increased with advancing age until the end of the 6^th ^decade of life, and decreased thereafter (p < 0.001). BMI values were abnormally high (> 25 Kg/m^2^) in nearly 86% of the total study population (1612 of 1890 women), and slowly and progressively increased with age, except in the 80+ decade (p < 0.001). Consistent with previous reports, QUS T-scores decreased significantly during aging (p = 0.01). SI values measured by QUS remained unchanged in pre- and postmenopausal women younger than 50 years of age, but decreased after the 6^th ^decade (p = 0.001; Figure 2). There was a negative correlation between SI values and age in all groups independent of activity status (p = 0.001 [r = -0.4], p = 0.001 [r = -0.6], and p = 0.001 [r = -0.5] for sedentary, moderately active, and active women, respectively). The frequency of physical activity across age groups in the Greek female population studied here (n = 1890) was extremely low. Only 6% took part in two 20-30-minute sessions of athletic activity per week and only 5% exercised systematically (three or more weekly sessions); the remainder (89%) led sedentary lives. Few postmenopausal women reported alcohol or tobacco use, and the amounts reported were generally low and would not be expected to be physiologically important. In addition, 43% of premenopausal women were smokers. As detailed in Figure 4, the amount of dietary calcium intake in the total population was inadequate.

### Association of menopausal status with anthropometric characteristics, QUS parameters and physical activity

Table 2 presents the numbers of participants in each activity group and percentages by menopausal status and age category. For reasons of homogeneity, premenopausal women in the age decade 50-59 years were not included in the analysis. As noted above, the overall activity level of this study population was low. Among premenopausal women, 9% were systematically active, 20% were moderately active, and 71% did not participate in physical activities; there were no age-related differences in activity level in this group. Furthermore, there was a decline in physical activity between active postmenopausal women in the 50-59 (5.3%) and 60-69 (1.70%) age-decade groups (p = 0.05); this trend toward reduced activity continued until the late 80 s but the difference was not significant compared with the activity level of the 60-69 age group. BMI increased after menopause until the end of the 6^th ^decade of life and decreased after the 7^th ^decade (p = 0.011). Similar to the total study population, approximately 86% of sedentary and moderately active women across all age decades (except 20-39-year-old premenopausal women) as well as active women aged more than 60 years had increased BMI values. If 86% of the total population had increased BMI values, certain subgroups must have BMI values that average (at least slightly) higher than this to balance out the exceptions noted (20-39-year-old premenopausal sedentary and moderately active women, and active women under 60.) BMI was negatively correlated with the duration of physical activity in premenopausal women in the 40-49 age group (p = 0.05, r = -0.4) and in all postmenopausal groups (p = 0.001, r = -0.6). In the < 50 age group (n = 446), the only significant difference in QUS T-scores noted was between the approximately 9% (41/446) of women who were systematically active and the 71% (317/446) who were sedentary (p < 0.029). Further analyses of individuals in the 40-49 age decade, which included both premenopausal (n = 120) and postmenopausal (n = 221) women, showed no additional differences in QUS parameters. Values of the SI parameter in sedentary, moderately active, and physically active women were 84.2 ± 18.2, 88.2 ± 18.6, and 93.1 ± 19.2, respectively. A comparison of SI values in sedentary premenopausal (95.5 ± 17, n = 371) and postmenopausal (80.8 ± 17, n = 1257) women, moderately active premenopausal (99.8 ± 15, n = 46) and postmenopausal (80.5 ± 17, n = 70) women, and systematically active premenopausal (104 ± 18, n = 24) and postmenopausal (85 ± 15, n = 34) women showed significant differences among premenopausal groups (p = 0.016) and between sedentary and active postmenopausal women (p = 0.039). Independent of menopausal status, the difference in SI values between active and sedentary women was highly significant (p = 0.001), but that between sedentary and moderately active women was not (p = 0.065). According to the adjusted r^2 ^values obtained by a stepwise, forward, multiple-regression analysis of the SI variable with age, weight, height, and BMI (data not shown), height appeared to be the least important factor for SI in all groups.

### Calcium intake and effects on QUS T-score

**Figure 5:**
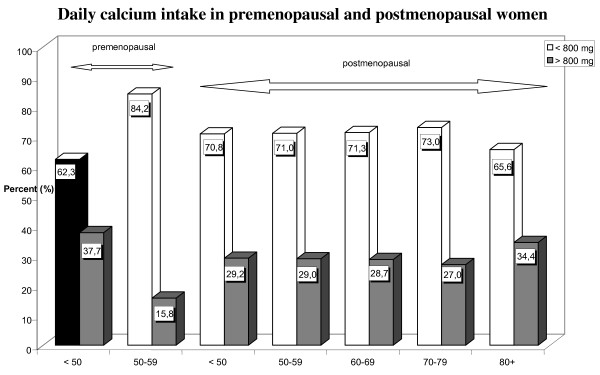
**Daily calcium intake in premenopausal and postmenopausal women**. This figure depicts the daily amounts of dietary calcium intake (and percent) according to menopausal status using 800 mg daily as cut off point.

Figure 5 shows the amounts of dietary calcium intake according to menopausal status. Calcium consumption in most groups, like that in the study population as a whole, was inadequate. Fewer than one third of premenopausal and postmenopausal women received more than 800 mg calcium daily, and fewer than 16% of premenopausal women in the 50-59 age decade (n = 38) consumed more than 800 mg calcium daily.

**Figure 6:**
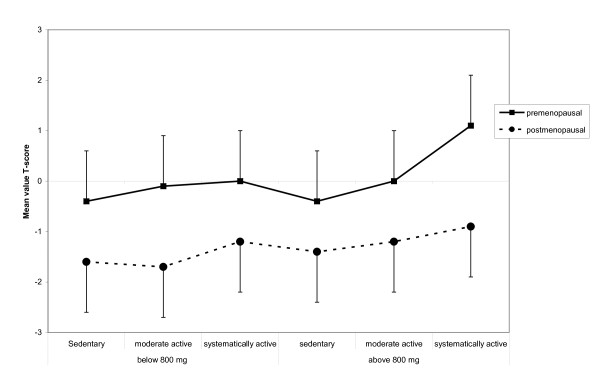
**Synergy between physical activity and dietary calcium intake in women consuming calcium amounts greater than 800 mg/day**. This graphic depicts mean QUS T-score values calculated according to daily calcium intake in all premenopausal and postmenopausal activity groups using 800 mg daily as cut off point (for statistical significant values see text).

QUS T-score were then calculated according to daily calcium intake in all premenopausal and postmenopausal activity groups using 800 mg daily calcium as cutoff point. As shown in Tables 3 and 4 and Figure 6, premenopausal women who were systematically active and consumed more than 800 mg calcium daily had significantly higher QUS T-scores compared with all other activity groups (p < 0.05). Among systematically active premenopausal women who received more than 800 mg calcium per day, this difference was separately significant verses sedentary (p = 0.028) and moderately active (p = 0.04) women. In contrast, postmenopausal women showed no difference in QUS T-scores regardless of the amount of daily calcium intake.

## Discussion

The aim of this study was to investigate the relationship between physical activity and dietary calcium intake, and calcaneal QUS parameters in Greek women. The results reveal a positive association between QUS and both lifestyle parameters. To our knowledge, this is the first study to investigate physical activity and its association with QUS parameters and dietary calcium intake in a Greek population of premenopausal and postmenopausal women. The power of our study lies in the large number of participants. Our findings also create a database of anthropometric metrics and ultrasound measurements of the calcaneus in the Greek female population.

We found that age and menopausal status were associated with QUS indices. These results are in agreement with those of previous cross-sectional studies that demonstrated substantial decreases in ultrasonic parameters with aging, particularly during the period after the menopause [33,34]. In the context of osteoporotic risk, body weight is believed to act positively on bone. There is evidence that larger body mass imposes a greater mechanical loading on bone that results in an increase in bone mass to accommodate the greater load. It has been proposed that increases in adipose tissue that occur with increasing BMI in postmenopausal women result in increased estrogen production, osteoclast suppression, and a resultant increase in bone mass [35]. Postmenopausal women in the current study had increased body weight and BMI values, in contrast to very old ladies. A European study found that more than 70% of Greek women aged more than 18 years have an abnormal BMI (> 25 kg/m^2^), making the Greek population among the most overweight in the European Union [36]. In the current study, we found that an even larger percentage (85%) of women were overweight. We also found that women in older age groups had lower values of QUS parameters independent of abnormal BMI values, suggesting that aging is a stronger predictor of bone status than BMI. Weight gain may be caused by reduced physical activity, increased calorie intake, or a loss in lean muscle; in most cases, all three factors likely contribute. In this study, women started gaining weight a few years before the menopause during the peri-menopausal period, when estrogen levels start to decline. In our population, BMI increased slowly during aging until the end of the 6^th ^decade owing to an increase in body weight and a decrease in height. Aging is associated with anatomical changes, including a gradual loss of bone and a progressive reduction in muscle mass, that together with a decline in cardiovascular fitness lead to physical impairment [37,38]. This concept is evidenced in our results, which showed that fewer than 1.5% of women remained systematically active after 60 years of age, and identified a large subgroup of inactive women (n = 1041) who are in need of innovative intervention strategies aimed at reversing the decline in lean tissue and increase in adiposity.

DXA predicts fracture risk and is considered the "gold standard" for the diagnosis of osteoporosis [39]. Although DXA is currently the most widely used bone densitometry method, access to DXA in some countries is limited [40]. In Greece, DXA is the most widely used method for measuring bone density; other methods, such as volumetric quantitative computed tomography (vQCT), QUS, and peripheral QCT, are rarely used. Greece, which is divided into 49 counties, has a total of 367 DXA units, of which 172 (46.86%) are located in the capital city, Athens [41]. However, DXA is not an optimal tool for population screening. As an alternative, we used QUS to screen populations, demonstrating our ability to measure bone status in the community using this noninvasive and less time-consuming method, which relies on a relatively inexpensive, radiation-free, portable device [42]. Our experience highlights the feasibility of using QUS as an alternative means to assess bone mass in clinical practice. Several large, cross-sectional studies of European and Asian populations, most of which have used the Lunar Achilles system, have generated normative data for ultrasound measurements of the calcaneus [43-46]. Our study shows an appreciable reduction in ultrasound-measured SI values and T-scores with age after menopause in a Greek population, whereas these values showed almost no decline with age in premenopausal women. These results are in agreement with other reports on age-related changes in os calcis QUS using the same device [43]. The 2002 Eurobarometer survey on physical activity, the most recent survey of the European population of its kind, included questions on the frequency and duration of vigorous activity, moderate activity, and walking [47]. This survey differed from our study methodologically because it: a) included both men and women; b) was not designed to investigate bone mass-promoting activities; and c) used a different questionnaire. The 2002 Eurobarometer survey used the "International Physical Activity Questionnaire", which allows a cross-cultural comparison of physical activity parameters. We, on the other hand, used a modification of the MEDOS questionnaire adapted to the Greek population. Despite these differences, the proportion of adults estimated to engage in regular physical activity were similar in the two studies: in the 2002 Eurobarometer, only 3.5% of the survey population undertook vigorous activities more than 2 hours per week, whereas in our study, only 5% of the Greek female population undertook such vigorous activities.

The os calcis, consisting of approximately 90% trabecular bone in the region measured by QUS, has a central position in supporting body weight. This is the skeletal site where ground-reaction forces applied with every heel-strike during exercise are maximal, and thus might be an appropriate site for evaluating the effects of integrated physical activity on bone [48]. Heinonen et al. confirmed this using DXA, showing that the response of the os calcis to physical activity was greater than that of the femur during an 18-month supervised training period and a subsequent 8-month follow-up unsupervised training period [49]. Cheng et al. assessed the effect of foot length and width on QUS variables measured using two Food and Drug Administration-cleared QUS devices--the Sahara (Hologic) and the Achilles+ (Lunar)--and found that after controlling for foot size, the correlation between QUS and BMD variables became stronger for both devices, especially for SOS [50]. Not all types of physical activities that provide bone loading to the skeleton produce bone mass benefits. Some activities (for example, a progressive jogging program) charge and stimulate adaptation of the cardiovascular system, but do not stimulate an adaptive bone response that would increase bone density [51]. There are also activities that provide bone loading at one site of the body, but not at other sites. The osteogenic effects of exercise should be specific to the anatomical sites where the mechanical strain occurs [52]. The most common types of physical activities (e.g., gardening, swimming) use many muscles but do not involve targeted bone loading, and therefore do not produce loads heavy enough to exceed the load threshold on bones achieved by usual daily activities [53,54]. The duration of the physical activity is also important; up to 2 hours per week is considered to positively affect bone mass maintenance [55-60]. Systematically active premenopausal and postmenopausal women in this study had significantly higher values of QUS parameters than their sedentary and moderately active counterparts. These results are consistent with those of previous studies showing a positive association of QUS parameters with higher levels of physical activity in women of various ages, and suggest the importance of physical activity for bone health throughout life [35,44,61-64]. Yamaguchi et al. reported that physical activity influences bone characteristics assessed by QUS [23]. Jones et al. examined the influence of brisk walking on QUS indices in a group of 40 formerly sedentary women and found increased values of QUS in walkers but a decrease in controls [65]. The statistically significant difference in QUS T-score that we found here between sedentary premenopausal women and those who exercise systematically may suggest that vigorous physical activity is a regulator of bone status during premenopausal years. Premenopausal women usually perform more intensive activities. For women in this group, high-impact exercise has been suggested to be the most effective regimen, with the gains induced being maintained after intervention. Vainionpaa et al. have shown a significant correlation between the intensity of exercise and changes in BMD [66]. Although the numbers of women in the different activity groups were not similar in our study, T-score and SI values were higher in systematically active women compared with the less active groups, independent of menopausal status. This finding can be explained in two ways. Either the physical activity associated with more than three 20-30-minute sessions per week protects against osteoporosis, or this subgroup included women who were well informed about the benefits of exercise in general health and performed specific bone-promoting activities as a part of their general activity program. Alcohol and smoking were not found to influence bone status in postmenopausal Greek women. This is in agreement with the results of previous studies and reflects the generally high prevalence of non-smokers and non-drinkers in our study population.

Calcium is an important nutritional factor for bone health. A review by Heaney of 139 reports investigating the role of calcium on bone mass concluded that calcium has positive effects on bone mass throughout life [67]. Another retrospective study also demonstrated a significant positive relationship between dairy product consumption in adolescence and BMD in young adult women [68]. The recommendations for daily intake of calcium range from 500 to 1500 mg/day in adult women, although studies suggest that the intake should be at least 1300 mg/day [69-72]. It is not entirely clear why we observed a relative lack of an effect of calcium intake on QUS measurements at the heel, but consistent with other epidemiological studies, we found an effect of high calcium intake [73]. Friedlander et al. showed that calcium supplementation neither enhanced exercise-induced bone benefit nor increased bone mass in the absence of exercise. This study indicated that over a 2-year period, a combined regimen of aerobics and weight training had beneficial effects on BMD and fitness parameters in young women. However, the inclusion of daily calcium supplementation produced no additional benefits [74]. Furthermore, in a review on the subject, Specker concluded that physical activity was beneficial only with a daily calcium intake of at least 1000 mg, and the beneficial effect of a high calcium intake was only evident in physically active groups [19]. The former finding is in line with our results, which showed a synergy between physical activity and dietary calcium intake in women consuming calcium amounts greater than 800 mg/day. Specifically, we found that only systematically physically active women had higher QUS T-scores than sedentary women. Conversely, women who consumed less than 800 mg calcium per day showed no difference in QUS T-score independent of activity level (Figure 6).

We recognize that there are limitations to our approach to the "measurement" of physical activity. First, physical activity is a difficult metric to obtain using a questionnaire, which measures perceptions that may be distorted. Thus, questions alone are probably not capable of detecting significant differences in activity among individuals. Second, the women included in our study were, on average, moderately active, with few engaging in high levels of physical activity. As a result, the variance associated with physical activity was more restricted than the variance associated with other variables such as calcium intake. It is clear that measurement by questionnaire, while expedient, has its limitations. Notwithstanding these limitations, it would be premature to discount the importance of physical activity to bone density. In future studies, activity diaries and motion sensors might be used to supplement the information obtained by questionnaire.

## Conclusions

Our results suggest that physical activity and adequate calcium consumption synergize to induce bone gains. Although calcium was measured at a single time point in our study and may thus not reflect lifelong calcium intake, the resulting insights were sufficient to highlight the synergy between physical activity and adequate calcium consumption. In combination with other curative interventions, it is important to inform and educate women regarding bone-promoting physical activities to maximize bone density benefits.

## Abbreviations

SI: stiffness index; QUS: quantitative ultrasound; BMD: bone mineral density; DXA: X-ray absorptiometry; BMI: Body Mass Index; SOS: speed of sound; BUA: broadband ultrasonic attenuation; ANOVA: analysis of variance; vQCT: volumetric quantitative computed tomography.

## Competing interests

The authors declare that they have no competing interests.

## Authors' contributions

YD conceived of the study, participated in the design, coordinated the study, and drafted the manuscript, IP participated in structuring the manuscript, GT coordinated the study, AG performed the statistical analyses and GL participated in the design and organization of the manuscript.

**All authors read and approved the final manuscript**.

## Pre-publication history

The pre-publication history for this paper can be accessed here:

http://www.biomedcentral.com/1472-6874/10/12/prepub

## Supplementary Material

Additional file 1 **Greek Osteoporosis Screening Study Questionnaire**. The questionnaire, consisting of questions in Greek, was divided into four sections: the first part included women's sociodemographic characteristics; the second part included women's menopausal status at the study time, the third part was focused on risk factors, and in the last section women answered questions about quality of life (social life, experienced changes in the body). Click here for file
