# Supplementary material for: Association of physical exercise and calcium intake with bone mass measured by quantitative ultrasound
Source: BMC Womens Health. 2010 Apr 7;10:12. doi: 10.1186/1472-6874-10-12 (PMC2858094; doi:10.1186/1472-6874-10-12)
Supplement: Additional file 1 — Greek Osteoporosis Screening Study Questionnaire. The questionnaire, consisting of questions in Greek, was divided into four sections: the first part included women's sociodemographic characteristics; the second part included women's menopausal status at the study time, the third part was focused on risk factors, and in the last section women answered questions about quality of life (social life, experienced changes in the body). [file 1472-6874-10-12-S1.DOC]

Additional file 1

Greek Osteoporosis Screening Study Questionnaire

AREA CODE :

***1^st^ part***

1. Name: ........................................................ patient code

2. Sex:

Female Male

3. Age: ........................

4. Height: ........................

5. Weight: ........................

6. Occupation: ........................

26. What is your flesh color?

Blond, fair, mild dark, dark

45. Do you wear conservative dresses (black clothes)?

Yes No

***2^nd^ part***

37. Are you at menopause?

Yes No

38. Age at menopause: ........................

39. Number of pregnancies: ........................

40. Number of children: ........................

***3^rd^ part***

27. Has either of your parents been diagnosed with osteoporosis?

Yes No

28. Has either of your parents broken a bone after a minor bump or fall?

Yes No

29. Have you broken a bone after a minor bump of fall?

Yes No

10. Have you ever been measured with a bone density scan for osteoporosis?

Yes No

11. Are you smoking?

Yes No

13. Are you smoking more than 20 cigarettes in a day?

Yes No

14. Do you regularly drink alcohol (all types) more than 2 glasses in a week?

Yes No

15. Do you drink more than 2 cups of coffee in a day?

Yes No

17. Do you drink or eat at least a glass of milk or yoghurt every day?

Yes No (if no, how often in the last year)

18. Do you eat at least a piece of cheese (30 g) every day?

Yes No (if no, how often in the last year)

19. Do you eat at ice-cream?

Yes No (if yes, how often in the last year)

20. Have you ever taken osteoporosis therapy?

Yes No

21. Have you ever taken corticosteroids more than 3 months?

Yes No

21. Which of the following chronic disease/s do you have?

Diabetes Hypertension Thyroid disease Renal disease Others

22. Which of the following medication/s of you use?

Thiazides, Anti-hypertensive, Warfarin, Thyroxin, Statins, Antiacids

24. Do you walk at least for 10 minutes many times during the week?

Yes No

25. Do you do gardening at least 3 hours per week?

Yes No

***4^th^ part***

23. Do you generally stay at home, watching TV or reading?

Yes No

30. Have you lost more than 3 cm in height?

Yes No

31. Do you have kyphosis?

Yes No

32. (if yes 30 and / or 31) Are these changes affecting you psychologically?

Yes No

The questionnaire, consisting of questions in Greek, was divided into four sections: the first part included women’s sociodemographic characteristics; the second part included women’s menopausal status at the study time, the third part was focused on risk factors, and in the last section women answered questions about quality of life (social life, experienced changes in the body).
